# Multi-modal-analgesia for pain management after Hallux Valgus surgery: a prospective randomised study on the effect of ankle block

**DOI:** 10.1186/1749-799X-2-26

**Published:** 2007-12-18

**Authors:** Ibrahim Turan, Hamid Assareh, Christer Rolf, Jan Jakobsson

**Affiliations:** 1 Karolinska Institutet, Foot & Ankle Surgical Centre, Stockholm, Sweden; 2 Sheffield Centre of Sports Medicine, University of Sheffield, UK

## Abstract

**Background:**

Pain and emesis are the two major complaints after day case surgery. Local anaesthesia has become an important part of optimizing intra and post-operative pain treatment, but is sometimes not entirely sufficient. The aim of the present study was to study the effect of adding an ankle block to a multi-modal analgesic approach on the first 24-hour-need for rescue analgesia in patients undergoing elective Hallux Valgus surgery.

**Type of study:**

Prospective, randomized patient-blind study comparing ankle block with levo-bupivacaine, lidocaine and Saline placebo control.

**Methods:**

Ninety patients were studied comparing ankle block (15 cc) using levo-bupivacaine 2.5 mg/ml, lidocaine 10 mg/ml or placebo (saline) on day-case elective Hallux Valgus surgery, supported by general anaesthesia in all cases. Primary study endpoint was number of patient's requiring oral analgesics during the first 24 post-operative hours.

**Results:**

Ankle block had no effect on need for rescue analgesia and pain ratings during the 1^st ^24 postoperative hours, there was no difference seen between placebo and any of the two active local anaesthesia studied. The only differences seen was that both lidocaine and levo-bupivacaine reduced the intra-operative need for anaesthetic (sevoflurane) and that levo-bupivacaine patients had a lower need as compared to the lidocaine patients for oral analgesics during the afternoon of surgery.

**Conclusion:**

Adding a single shot ankle block to a multi-modal pain management strategy reduces the need for intra-operative anaesthesia but has no major impact of need of rescue analgesics or pain during the first 24-hour after surgery.

## Background

Multi modal pain management has become standard of care especially in day case surgery [1]. Local anaesthesia applied prior to incision has been shown to have positive effects on the postoperative course [2]. However, in many cases this is not sufficient to relieve pain after surgery [3]. The impact of adding a peripheral nerve block to local wound infiltration has not been studied in Hallux Valgus surgery. Hypothetically it could decrease postoperative discomfort and pain, allowing the patient to mobilise faster and with less rescue medication.

The aim of the present study was to evaluate the effects of adding a single shot ankle block to a routine multi-modal pain strategy for the management of Hallux Valgus surgery performed under general anaesthesia.

## Methods

After informed consent 90 healthy America Society of Anesthesiology functional classes 1–2 patients undergoing elective Hallux Valgus surgery ad modum Turan were studied [4]. The study protocol, a prospective randomised study of the effects of a single shot ankle block on the need for rescue analgesia during the first 24 hours following surgery was approved by Karolinska Institutet's local ethical committee. The patients were randomised into three groups by closed envelope technique;

Group A had 15 cc levo-bupivacaine 2.5 mg/ml

Group B had 15 cc lidocaine 10 mg/ml

Group C Placebo Control had 15 cc of saline

The performance of the ankle block was; "the posterior tibial nerve" by 5 cc posterior to the medial malleoli, "the peroneal nerve, superficial and deep" by 6 cc on dorsum of the foot and "the sural nerve" by 4 cc posterior to the lateral malleoli.

All patients followed the routine pre and postoperative protocol of our institution. They were asked to refrain from eating for 6 hours and drinking for 2 hour prior to surgery.

After establishment of an intravenous line all patients were given 8 mg betamethasone, 0.3 mg alfentanil and 30 – 50 mg propofol. After sedation the patients received the ankle block in accordance with the randomisation in a single blinded fashion.

All patients also had 10 cc of 10 mg/ml lidocaine subcutaneous around the surgical incision given by the orthopaedic surgeon (IT) under sterile conditions right prior to incision. A general anaesthesia was administered by means of sevoflurane in oxygen/air by mask. Sevoflurane was titrated according to clinical needs by the attending anaesthetist (JJ). Sevoflurane concentrations were continuously monitored by side-stream gas analysis. Peak end-tidal and mean end-tidal sevoflurane concentrations were recorded and used for evaluation of need for anaesthesia.

Immediately after surgery anaesthesia was discontinued and the patients moved to the recovery area, if fully awake and alert possibly bypassing conventional recovery room stay. After arrival in recovery area all patients had an initial oral dose of etoricoxib 120 mg and a loading dose of 30 mg/kg paracetamol.

Patients were discharged when awake, and ambulant with minimal and acceptable levels of subjective pain (VAS < 4).

At discharge all patients were provided with take-home analgesic medication; Etoricoxib 120 mg, paracetamol 1 gr. and dextropropoxyphene 100 mg. All patients were provided with oral and written instruction about pain management at home they were informed to take:

• Regularly etoricoxib 120 mg once daily

• Additional paracetamol 1 gr. oral when needed (up to four grams daily), as initial pain therapy

• Additional dextropropoxyphene 100 mg oral if paracetamol had reviled pain within 30 minutes

They were instructed to take patacetamol and dextropropoxyphene not by routine, but only when needed.

Patients were instructed to fill in a protocol for the first postoperative day; 1^st ^24 post operative hours, need for any pain medication; paracetamol and dextropropoxyphene, as well as pain ratings at a verbal pain scale; no pain, little pain, pain, severe pain [5].

### Statistics

Patients' demographics are presented as mean and standard deviation. All results are given as median and range. None-parametric Chi-square test or Mann-Whitney U-test when appropriate studied differences between groups. The number of patients (30 in each group) was determined by a pre-study power analysis based on the findings in a pilot study in our institution; need for additional analgesics in the control (placebo) group of 70% and a clinical relevant absolute reduction in patient taking additional analgesics of 30%, corresponding to clinical significant effect of an ankle block being that only 40% of patients needing additional analgesics during the first postoperative 24 hours with a power of 90 % at p < 0.05. All statistics were made in StatView™ on a Macintosh computer system.

## Results

There was no significant difference in patient demographics Table 1. Nine procedures were a combination of a Hallux Valgus ad modum Turan and a hammertoe procedure. All surgery and anaesthesia was uneventful and no complications or adverse effects were noticed. Duration of procedures was also the same in all three groups; mean duration of surgery (knife to skin to closed wound) was 12 (9–22) minutes. The need for sevoflurane was higher in the placebo group of patients (end tidal gas concentrations were significantly lower in the levo-bupivacaine and lidocaine patients) otherwise no differences could be seen intra-operatively or during the early postoperative period, until discharge (Table 2). Emergence was rapid and all patients were safely "fast-tracked", by-passing the regular recovery room, into a step-down area. Recovery times, time to allowing drinking and intake of oral medication and time to be eligible for discharge were also the same in all three groups. All patients were discharged home safely within 60 minutes from reaching the recovery area/step-down unit. No patient required any rescue analgesia or anti-emetics during the recovery period.

**Table 1:** Patient's demographics

	**Lidocaine **(n = 30)	**Levo-bupivacaine **(n = 30)	**Placebo **(n = 30)
***Sex (male/female)***	6/24	1/29	4/26
***Age (year.)***	45 ± 15	50 ± 12	46 ± 13
***Weight (kg)***	69 ± 12	65 ± 98	70 ± 17
***Surgery HV/HV + "hammer toe"***	28/2	26/4	27/3

HV Hallux Valgus

**Table 2:** Intra and post operative observations

	**Lidocaine **(n = 30)	**Levo-bupivacaine **(n = 30)	**Placebo **(n = 30)
***Surgery HV/comb***	28/2	26/4	27/3
***Duration of surgery (min.)***	12.4 ± 2.1	12.4 ± 2.7	11.9 ± 1.6
***Propofol (mg)***	104 ± 36	106 ± 38	115 ± 31
***Et Sevo peak (%) ***	1.38 ± 0.55	1.42 ± 0.40	1.85 ± 0.67 **
***Et Sevo mean (%) ***	0.99 ± 0.35	1.03 ± 0.26	1.43 ± 0.45 **
***OAAS 5 (min.)***	8 ± 3	8 ± 3	8 ± 3
***Discharge (min.)***	40 ± 8	40 ± 9	42 ± 9

** p < 0.01 ANOVA

HV Hallux Valgus

Et Sevo end tidal concentration of sevoflurane

OAAS Observer Assessment of Alertness Scale (5 awake, 0 asleep not responding to noxious stimulation).

The number of patients that needed further oral analgesics during the first 24 postoperative hours was 21, 9 and 17 out of 30 for the lidocaine, levo-bupivacaine and placebo group of patients respectively with no significant difference between either of the two active treatments as compared to placebo, Table 3. Levo-bupivacaine did not significantly increase the number rescue analgesia-free patients as compared to placebo (p < 0.052) a significant difference was seen, when analysed separately, between levo-bupivacaine and lidocaine groups of patients (p < 0.0036). When levo-bupivacaine was compared to the combined group of patients' lidocaine and placebo control a significant difference was also noticed (p < 0.0058).

**Table 3:** Primary Outcome need for rescue analgesics during the first 24 hours

	**Lidocaine **(n = 30)	**Levo-bupivacaine **(n = 30)	**Placebo **(n = 30)
***Paracetamol***	15	4	12
***Detropropoxyphene***	6	5	5
***No analgesia***	9 *	21	13

Number of patients in each group

p < 0.05 as compared to the levo-bupivacaine group only

**Figure 1:**
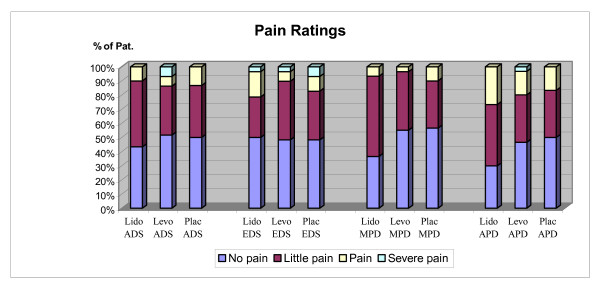
**Pain ratings in each group, percentage of patient**. Lido = lidocaine, Lev = levo-bupivacaine, Plac = placebo ADS = afternoon day of surgery, EDS = evening day of surgery MPD = morning post operative day 1, APD = afternoon post operative day 1.

Pain ratings were overall low, and waste majority of patients made pain ratings as no or little pain. Nine patients (10%) made a rating of *painful *during afternoon and 10 (11 %) in the evening on the day of surgery. In the morning and afternoon on the 1^st ^postoperative day 6 (7%) and 18 (20%) patients respectively scored the intensity as *painful*. Five patients made a rating of *severe pain *on 1 occasion and 1 patient on 2 occasions, afternoon and evening day of surgery. No difference in pain ratings was seen between the groups figure 1.

No complications or adverse events were noticed during the follow-up period.

## Discussion

The interpretation of our results should be done with caution, as the study design is complex. Our study is negative; we could not see any statistical significant difference between the levo-bupivacaine or lidocaine block and placebo with regard to our primary study endpoint; number of patients that needed oral pain medication during the first 24 post operative hours. Interestingly though is that a post hoc separate comparison between lidocaine and levo-bupivacaine revealed significantly less patients taking any further pain medication after a levo-bupivacaine block as compared to the lidocaine block. Also when the patients that received the long-acting local anaesthetic levo-bupivacaine was compared to the combined group of patients either given the short acting lidocaine block or placebo control a significant difference was observed in favour for the levo-bupivacaine block. No difference was however found in the need for "further dextropropoxyphene rescue medication". Also pain ratings were similar between the all three groups studied; the addition of 15 cc of lidocaine 10 g/ml or levo-bupivacaine 2.5 mg/ml did not affect pain ratings during the first 24 postoperative hours as compared to placebo. We found also that adding both 15 cc of levo-bupivacaine 2.5 mg/ml or lidocaine 10 mg/ml as an ankle block prior to surgery to a multimodal pain management during Hallux Valgus surgery decreased the need for intra-operative anaesthesia by decreasing the concentration of sevoflurane needed; however no difference in emergence/recovery time characteristics was noticed.

The use of local anaesthetic is not a new concept but a basic component used in clinical practice all over the world. It is important to take into account that all our patient had local anaesthesia, lidocaine, applied locally by the surgeon before skin incision. In order to achieve a rapid onset lidocaine has been the tradition of use in our institution for locally applied anaesthesia. It would of course be of interest to study the effects on postoperative pain by changing to or adding a long acting local anaesthetic to the locally applied block. Already in 1985 Porter and Davis showed that injecting a long acting local anaesthetic into the pseudoartrosis immediately after skin closure gave better postoperative pain relief as compared to placebo after Keller's procedure [6]. The hypothesis of the present study was that adding an ankle block with a long lasting local anaesthetic to the routine of local anaesthesia with lidocaine would enhance postoperative analgesia and reducing the need for further analgesics during the first postoperative 24 hours. Similar to what we previously found for intra-articular injection in knee arthroscopy [7]. We also hypothesized that the adding a short-acting block lidocaine ankle block would provide the intra-operative effects but without major contribution on protracted postoperative pain course and the need for postoperative analgesics after discharge. We used the technique for providing a classic ankle block that was described already 1983 by Sarrafian et al [8]. A seemingly safe, simple and cost effective approach; looking at a 3 puncture ankle block with a plain classic lidocaine 1 % or long lasting low concentration local anaesthetic, levo-bupivacaine 2.5 mg/ml. Lately more sophisticated techniques have been described. Casati and co-workers studied sciatic nerve blocks and showed reassuring result from that technique [9]. Reinhart et al combined lidocaine and ketorolac and found the locally applied combination more effective than lidocain used for the ankle block and intravenous ketorolac [10]. Continuous infusion of long acting local anaesthesia has also been described in Hallux Valgus surgery with positive results [11,12]. It is important to recognise that adding an ankle block is not entirely uncomplicated; it is time consuming and entails both cost and risk. Both lidocaine and levo-bupivacaine have a low toxicity; still injecting 15 cc close to ankle arteries and veins is associated with the potential risk for intravascular injection and systemic toxicity.

It should indeed be acknowledged that our patients all received a multi-modal analgesic regime. All patients received both bethametasone and a small dose of alfentanil right before induction of anaesthesia. Romunstad et al has convincingly shown the analgesic effects of steroids [13], improving not only pain but also overall patient satisfaction. Furthermore, all patients received etoricoxib in maximal recommended dose immediately after surgery in combination with a loading dose of paracetamol. The use of NSAIDs and Coxibs has been debated because of their theoretical risk of causing impaired bone healing. The positive effect of coxibs in fore foot surgery has, however been shown both by Pollak and Desjardines [14,15]. The clinical evidence for any major impact from short term NSAID/Coxib therapy on the bone healing is not well documented [16]. And the use of NSAID/Coxib has been the routine at our department since long. The oral route has been advocated as equally effective to intravenous administration and has been the standard of care in our institution over many years [17]. The 30 mg/kg dose Paracetamol should guarantee an early therapeutic plasma concentration [18].

One may argue that the patients were given an extensive peri-operative analgesics regime and that in orthopaedic day surgical procedures such as knee arthroscopy the need for postoperative pain medication is not that extensive [7]. It is recognized that Hallux Valgus surgery is associated with far more extensive postoperative pain than for example a partial meniscectomy [11]. The power of the study was based on the assumption that 70 % of the control group of patients would experience pain to that extent that intake of further analgesic after discharge was needed. We found a somewhat lower number, still 57 % of the control patients required further analgesic, and in the lidocaine group 70 % of patients required post discharge analgesics. Whether the slightly higher need for further analgesics in the lidocaine group is a sign of rebound pain is beyond the scoop of this study.

## Conclusion

When added to a multi-modal pain management a single-shot ankle block was not enough to show any significant difference in the need for further analgesic and did not change the pain ratings during the first 24 hours after elective Hallux Valgus surgery as compared to placebo. Adding an ankle block reduced however the intra-operative anaesthetic requirement and a levo-bupivacaine block provides similar beneficial intra-operative effects but reduced also the need for rescue analgesics during the first 24 hours as compared to the lidocaine block.

## Competing interests

There are no competing interests; this is a basic academic research initiative.

## Authors' contributions

JJ has had the main responsibility for the study and manuscript preparation.
